# Severe acute respiratory syndrome coronavirus 2 infection among healthcare workers in a tertiary public hospital in Curitiba, Brazil

**DOI:** 10.1590/0037-8682-0265-2021

**Published:** 2022-02-25

**Authors:** Regiane Nogueira Spalanzani, Gustavo Genelhoud, Sonia Mara Raboni, Sergio Monteiro de Almeida, Luciane Aparecida Pereira, Indianara Rotta, Barbara Maria Cavalli, Francielli Brusco Moreira, Carolina Lumi Tanaka Dino, Gislene Reche de Almeida Takahashi, Regielly Caroline Raimundo Cognialli, Beatriz Sanada Spiri, Lucas Bochnia-Bueno, Jaqueline Carvalho de Oliveira, Douglas Adamoski, Daniela Fiori Gradia, Ana Cláudia Bonatto, Roseli Wassem, Juliana Mazini Alves, Raquel da Silva Padilha, Vitor Jorge Woytuski Brasil, Bernardo Montesanti Machado de Almeida, Meri Bordignon Nogueira

**Affiliations:** 1 Universidade Federal do Paraná, Hospital de Clínicas, Laboratório de Virologia, Curitiba, PR, Brasil.; 2 Universidade Federal do Paraná, Hospital de Clínicas, Programa de Residência Multiprofissional em Atenção Hospitalar, Curitiba, PR, Brasil.; 3 Universidade Federal do Paraná, Departamento de Genética, Curitiba, PR, Brasil.; 4 Universidade Federal do Paraná, Divisão de Doenças Infecciosas, Curitiba, PR, Brasil.; 5 Universidade Federal do Paraná, Programa de Pós-Graduação Strictu Sensu em Tocoginecologia e Saúde da Mulher, Curitiba, PR, Brasil.; 6 Universidade Federal do Paraná, Programa de Pós-Graduação Strictu Sensu em Microbiologia, Parasitologia e Patologia, Curitiba, PR, Brasil.; 7 Universidade Federal do Paraná, Hospital de Clínicas, Serviço de Saúde Ocupacional, Curitiba, PR, Brasil.; 8 Universidade Federal do Paraná, Hospital de Clínicas, Serviço de Epidemiologia, Curitiba, PR, Brasil.

**Keywords:** Health personnel, COVID-19, Nosocomial transmission, Molecular diagnosis, SARS-CoV-2

## Abstract

**BACKGROUND:**

We aimed to describe the clinical characteristics of coronavirus disease 2019 (COVID-19) among healthcare workers (HCWs) in Curitiba, Brazil.

**METHODS:**

Upper respiratory samples from 1077 HCWs were tested for severe acute respiratory syndrome coronavirus 2 (SARS-CoV-2) using reverse transcription polymerase chain reaction from June 16, 2020 to December 9, 2020.

**RESULTS:**

Overall, 32.7% of HCWs were infected. The positivity rates in symptomatic and asymptomatic HCWs were 39.2% and 15.9%, respectively. Hospital departments categorized as high-risk for exposure had the highest number of infected HCWs.

**CONCLUSIONS:**

Early diagnosis and isolation of infected HCWs remain key in controlling SARS-CoV-2 transmission because HCWs in close contact with COVID-19 patients are more likely to be infected than those who are not.

Severe acute respiratory syndrome coronavirus 2 (SARS-CoV-2), the causative agent of the coronavirus disease 2019 (COVID-19), emerged in Wuhan, China, and rapidly spread worldwide. It was declared a pandemic by the World Health Organization (WHO) on March 11, 2020^1^. By March 2021, nearly 119.2 million people had been infected, with over 2.6 million deaths reported globally^2^.

Transmission occurs mainly through respiratory droplets, close contact with an infected person, or self-contamination after contact with SARS-CoV-2-contaminated surfaces^3^. In healthcare settings, transmission from patients to healthcare workers (HCWs) may also occur during aerosol-generating procedures. HCWs are continuously exposed to infection during their day-to-day activities^4^ ^-^ ^6^.

Although the WHO has recommended implementing safety protocols for HCWs, such as using proper personal protective equipment (PPE)^7^, HCWs have a high SARS-CoV-2 infection rate worldwide due to the shortage of PPE and the lack of training in infection prevention and control^6^. Transmission may also occur in non-medical areas of the hospital while speaking or eating without following appropriate prevention measures^8^. Considering that HCWs may potentially be infected because of their exposure to COVID-19 patients and other HCWs during work shifts, it is essential to identify infected HCWs to prevent the spread of SARS-CoV-2. Therefore, this study aimed to assess the frequency of SARS-CoV-2 infection among HCWs in a tertiary public hospital in Curitiba, Brazil, reference in assistance of COVID-19 patients, and evaluate the association between occupation, workplace, and presence of symptoms.

The study was performed between June 16, 2020 and December 9, 2020, at the Complexo Hospital de Clínicas/Universidade Federal do Paraná, a 650-bed tertiary public hospital that offers the largest number of beds for COVID-19 in Curitiba, Brazil. It provides 83 nursery beds and 82 ICU beds for the exclusive care of patients infected with COVID-19. According to the hospital protocol, all HCWs (including residents, physicians, nurses, physiotherapists, laboratory technicians, administration, and social workers) presenting symptoms consistent with COVID-19 infection, or those with high-risk exposure to infected persons, were referred to the occupational health clinic and, a respiratory sample (combined swab) was collected between the 3rd and 5th days of symptom onset to undergo SARS-CoV-2 reverse transcription polymerase chain reaction (RT-PCR) testing. Some HCWs were tested more than once, but in a different period during the study according to their symptoms or exposure. Combined swab samples from the oropharynx (OPS) and nasopharynx (NPS) were collected, stored in a viral transport medium (VTM), and transported to the virology laboratory.

Clinical and epidemiological information of HCWs was collected retrospectively from occupational health department records. The following information was obtained: quantitative RT-PCR (RT-qPCR) results, age, sex, occupation, workplace, previous contact with a patient, relative, or HCW with positive RT-qPCR, and presence of COVID-19 symptoms (e.g., fever, cough, dyspnea, asthenia, myalgia, coryza, sore throat, headache, ageusia or dysgeusia, anosmia or parosmia, ocular symptoms, diarrhea, nausea, and vomiting). 

The HCWs were stratified into three categories according to their presumed level of exposure to COVID-19: high, moderate, and low risk of exposure. The high-risk category included HCWs in immediate contact with COVID-19 patients, such as emergency units, internal medicine, semi-intensive care, intensive care, and infectious disease departments. The moderate-risk category included those who worked in medical and surgical departments with occasional contact with COVID-19 patients, while the low-risk category included administrative workers, social workers, hospital management, pharmacies, and other areas with no COVID-19 patient contact.

RNA extraction was performed using a Biopur Mini Spin Virus DNA/RNA Extraction Kit (Biopur, Bethlehem CT, USA), and amplification was performed using the BIOMOL OneStep/COVID-19 kit (IBMP, Curitiba, Brazil), according to the manufacturer's instructions. A 7500 Real-Time PCR System (Applied Biosystems, San Francisco CA, USA) was used to detect the nucleocapsid (N) and open reading frame 1ab, with human RNase P as an internal control gene. Samples with inconclusive results due to amplification of only one of those targets were subjected to a rapid molecular test using Xpert^®^ Xpress SARS-CoV-2 (Cepheid, Sunnyvale CA, USA), which targets the N2 and E genes for confirmation.

According to the frequency of positive tests, the categorical variables' evaluation was calculated using the chi-square test, adjusted odds ratios (ORs), and 95% confidence intervals (CI). Statistical analyses were performed using MedCalc® Statistical Software (version 19.8; MedCalc Software Ltd, Ostend, Belgium). HCWs with multiple tests were screened, and only one test from each was used for statistical analysis. We considered the first test performed, and if symptoms were reported for tests with the same result.

The Ethical Committee Boarding of the Complexo Hospital de Clínicas of Universidade Federal do Paraná approved the study (CAAE: 31687620.2.0000.0096).

Since the confirmation of the first case in Brazil on February 26, 2020, in São Paulo state, COVID-19 has spread to all regions of the country, deeply impacting health professionals who were not properly prepared for the handling of critically ill patients or trained in the use of PPE to prevent transmitted disease by aerosol. This crisis has worsened because of a shortage of PPE in the country. With the warning of the COVID-19 pandemic, the Complexo Hospital de Clínicas began to prepare to receive patients with suspected SARS-CoV2 infection. The preparation included the training of professionals for patient care, changes in the hospital's internal flows, development of guidelines to fight the pandemic, and maintaining the stock of PPE that was lacking throughout the country. Safety and work protocols were evaluated and reassessed daily by a committee of experts. Our professionals, both in the health and administrative areas, were instructed to keep up to date and follow clinical protocols and internal flows. There was no lack of PPE, but intense work was performed seeking in the rationalization of its use since the risk of shortage of these was real. On March 27, 2020, the first case of COVID-19 infection was registered at the Complexo Hospital de Clínicas.

Among the 1077 HCWs evaluated in this analysis, 352 (32.7%) tested positive for SARS-CoV-2. The clinical and epidemiological characteristics of the HCWs are shown in Table 1. The average age of the study population was 39.8 years, and 803 (74.5%) were women.

**TABLE 1:** Clinical and epidemiological characteristics of 1077 HCWs tested for SARS-CoV-2 in Curitiba, Brazil, from June 16, 2020 to December 9, 2020.

Characteristic	Total	Positive test		Negative test		OR	p value
	n	n	%	n	%		
	1077	352	32,7	725	67,3		
**Sex**							
Female	803	244	30,4	559	69,6		0,01
Male	274	108	39,4	166	60,6		
**Age Median (IQR)**							
Median (IQR)		40(33; 48)		39(32; 46)			0,06
**Occupation**							
Physiotherapists	44	16	36,4	28	63,6	1.85	0.20
Healthcare assistants^a^	518	179	34,6	339	65,4	1.71	0.08
Physicians	209	71	34,0	138	66,0	1.67	0.13
Nurses	196	58	29,6	138	70,4	1.36	0.43
Others^b^	42	12	28,6	30	71,4	1.30	0.65
Clerical workers, technicians	68	16	23,5	52	76,5	reference group	
**Presence of symptoms**							
Yes	775	304	39,2	471	60,8		< 0.0001
No	302	48	15,9	254	84,1		
**Reported contact with infected person**	302	118	39,1	184	60,9		0.01
**Comorbidities**							
Reported at least one comorbidity	175	54	30,9	121	68,8	0.91	0.64
Cardiovascular diseases	62	29	46,8	33	53,2	1.79	0.04
Hematological diseases	10	4	40,0	6	60,0	1.36	0.64
Autoimmune diseases	8	3	37,5	5	62,5	1.22	0.79
Metabolical/hormonal diseases	37	12	32,4	24	64,9	1.02	0.96
Rheumatological diseases	6	1	16,7	5	83,3	0.41	0.40
Respiratory diseases	46	5	10,9	41	89,1	0.25	0.002
Psychologic diseases	4	-	-	4	100,0	-	0.16
Infectious diseases	3	-	-	3	100,0	-	0.23
No comorbidity	328	108	32,9	220	67,1	reference group	

^a^ includes nursing technicians and assistants, laboratory technicians, biologists, nutritionists, and pharmacists. ^b^includes graduation students and workers of the hospital such as engineers, drivers, and others. HCW: health-care workers; IQR: interquartile range; SARS-CoV-2: severe acute respiratory syndrome coronavirus 2. The p values were calculated by chi-square test. For occupation, exact fisher test were used to compare every category with clerical workers, technicians.

These findings show a higher infection rate than those previously reported in other countries, such as 11%, 8.8, and 7,1% in Spain, Italy, and Turkey, respectively^4^ ^,^ ^5^ ^,^ ^8^. However, Buonafine et al.^9^ in São Paulo, Brazil, reported 42.4% confirmed COVID-19 infections among symptomatic HCWs, while our findings demonstrated a 32.7% positivity rate among symptomatic HCWs.

Physiotherapists (16, 36.4%) showed the highest SARS-CoV-2 infection rate in our study, followed by healthcare assistants (179, 34.6%), and nursing technicians and physicians (71, 34%). 

In the present study, the positivity rate in symptomatic HCWs was 39.2% (304/775) (p < 0.0001), while 48/302 (15.9%) of the positive HCWs were asymptomatic. The positivity rate among asymptomatic HCWs varies from 3.4% in Chile^10^ to 34.9% in China^11^. These findings highlight the importance of regular testing in high-risk areas for SARS-CoV-2 transmission and contact tracing optimization, which will allow for the early detection and isolation of positive HCWs, preventing transmission to other HCWs, patients, and those in the community. Although COVID-19 is more contagious when an individual is symptomatic, transmission may also occur during the pre-symptomatic incubation period of the disease, which is estimated to be between 2 and 10 days^8^ ^,^ ^12^. Considering this situation, early detection and isolation of asymptomatic HCWs is critical for preventing SARS-CoV-2 transmission.

Overall, 28% (302/1077) of the HCWs reported previous contact with an infected person, and the positivity rate was 39.1% (118/302) (p = 0.01). A total of 175 individuals out of 1077 HCWs (16.2%) reported at least one comorbidity. Of these, 54 (30.9%) tested positive for SARS-CoV-2, of which 29 (46.8%) had cardiovascular disease, 12 (32.4%) had metabolic and hormonal diseases, five (10.9%) had respiratory disease, four (40%) had hematological disease, three (37.5%) had autoimmune disease, and one (16.7%) had a rheumatologic disease. 

Anosmia or parosmia (92, 80.7%), ageusia or dysgeusia (35, 74.5%), and dyspnea (56, 69.1%) were the most frequent symptoms associated with a positive RT-PCR for SARS-CoV-2. These results were similar to those previously reported, where the strongest predictors of infection were dysgeusia and anosmia^5^. According to our results, the chance of a patient with anosmia or parosmia, ageusia or dysgeusia, or dyspnea to present positive RT-PCR results were 11.31, 6.56, and 5.30-fold greater, respectively, than those who did not report these symptoms (p < 0.0001). Other symptoms are shown in Table 2.

**TABLE 2:** Association between reported symptoms and RT-PCR test results among the 1077 HCWs tested for SARS-CoV-2 in Curitiba, Brazil, from June 16, 2020 to December 9, 2020.

Symptoms	Total	Positive test		Negative test		p value	OR	95% CI
	n	n	%	n	%			
Anosmia or parosmia	114	92	80.7	22	19.3	< 0.0001	11.31	6.95 - 8.39
Ageusia or dysgeusia	47	35	74.5	12	25.5	< 0.0001	6.56	3.36 - 2.81
Dyspnea	81	56	69.1	25	30.9	< 0.0001	5.30	3.24 - 8.65
Ocular symptoms	15	9	60.0	6	40.0	0.02	3.14	1.11 - 8.90
Fever	173	99	57.2	74	42.8	< 0.0001	3.44	2.46 - 4.81
Asthenia	121	66	54.5	55	45.5	< 0.0001	2.81	1.92 - 4.13
Myalgia	334	166	49.7	168	50.3	< 0.0001	2.96	2.26 - 3.88
Cough	352	171	48.6	181	51.4	< 0.0001	2.84	2.17 - 3.71
Diarrhea	134	55	41.0	79	59.0	0.03	1.51	1.05 - 2.19
Nausea	52	21	40.4	31	59.6	0.22	1.42	0.80 - 2.51
Coryza	395	153	38.7	242	61.3	0.001	1.53	1.18 - 1.99
Headache	280	98	35.0	182	65.0	0.34	1.15	0.86 - 1.53
Sore throat	243	57	23.5	186	76.5	0.0005	0.56	0.40 - 0.78
Vomiting	17	3	17.6	14	82.4	0.18	0.44	0.12 - 1.53

HCW: health-care workers; OR: odds ratio; CI: confidence interval; SARS-CoV-2: severe acute respiratory syndrome coronavirus 2. The p values were calculated by chi-square tests.

The proportion of positive cases according to the presumed level of exposure is shown in Table 3. The high-risk category was the most affected and revealed to be statically significant (p < 0.0001), in which 40% (171/428) of the HCWs tested positive for COVID-19, demonstrating that HCWs in direct contact with infected patients were more likely to be infected than other professionals who were not in direct contact. However, it is essential to note the high frequency of infection among HCWs from workplaces with moderate (29%) and low risk of exposure (23.5%). According to our results, the high frequency of positive tests in moderate and low-risk exposure categories, which includes departments with minimum or no contact with COVID-19 patients, suggests that transmission commonly occurs among HCWs, which has been reported in other studies^4^. In Figure 1, it can be observed that there was a higher frequency of positive tests in the high-risk group at first but decreased afterward as the professionals in the low-risk category were tested positive. When we evaluated the first two months (June - July) in comparison to the last two months (November - December), the frequency of low- and moderate-risk cases among all the positive cases increased from 32.56% to 61.54% (Fisher exact test p = 0.0143). It has been pointed out that clinical meetings, clinical handovers, lunch breaks, and shared facilities may lead to potential transmission between HCWs^4^ ^,^ ^6^ ^,^ ^8^. In addition, Çelebi et al.^8^ suggested that staying in the same personnel break room as an HCW without wearing a medical mask for more than 15 minutes, food consumption within 1 m of another HCW, and failing to maintain a social distance from an HCW, are significant risk factors for the transmission of SARS-CoV-2. In addition, a cohort study by Ran et al.^13^ showed that nosocomial transmission of SARS-CoV-2 was linked with prolonged work hours, particularly in high-risk departments, and suboptimal hand washing. However, in the low-risk category, workers in the hospitality, cafeteria, and cleaning departments showed a 20% (1/5) positivity rate for COVID-19 infection, which indicates that transmission may also occur outside of medical areas. Since contact is one of the main routes for SARS-CoV-2 transmission, good hand hygiene is considered one of the most important prevention measures for HCW-associated infections in and out of hospital settings.

**TABLE 3:** SARS-CoV-2 positive and negative RT-PCR tests according to the HCWs’ workplace and presumed level of exposure.

Level of exposure	Workplace	Total	Positive tests		Negative tests		p value	OR	95% CI
		n	n	%	n	%			
High	Semi-intensive care units	34	17	50.0	17	50.0	0.21	4	0.40 - 39.58
	COVID-19 units	187	89	47.6	98	52.4	0.22	0.36	0.39 - 33.1
	Emergency units	64	21	32.8	43	67.2	0.55	1.95	0.21 - 18.58
	Intensive care units	96	30	31.3	66	68.8	0.59	1.82	0.19 - 16-97
	Internal medicine	47	14	29.8	33	70.2	0.65	1.70	0.17 - 16.57
	**Total of high-risk exposure level**	**428**	**171**	**40.0**	**257**	**60.0**	**0.001**	**2.17**	**1.38 - 3.38**
**Moderate**	Surgical units	80	36	45.0	44	55.0	0.38	2.62	0.28 - 24.38
	Laboratory	47	14	29.8	33	70.2	0.65	1.70	0.17 - 16.57
	Non-COVID-19 medical areas	339	88	26.0	251	74.0	0.76	1.40	0.15 - 12.7
	Gynecology and obstetrics	51	12	23.5	39	76.5	0.85	1.23	0.13 - 12.09
	**Total of moderate-risk exposure level**	**517**	**150**	**29.0**	**367**	**71.0**	**0.21**	**1.33**	**0.85 - 2.07**
**Low**	Administrative, management, preventive management and social workers	104	25	24.0	79	76.0	0.83	1.27	0.14 - 11.85
	Pharmacy	23	5	21.7	18	78.3	0.93	1.11	0.10 - 12.3
	Hospitality, cafeteria and cleaning personnel	5	1	20.0	4	80.0	reference group		
	**Total of low-risk exposure level**	**132**	**31**	**23.5**	**101**	**76.5**	**exposure risk reference group**		

HCW: health-care workers; OR: odds ratio; CI: confidence interval; SARS-CoV-2: severe acute respiratory syndrome coronavirus 2; COVID-19: coronavirus disease 2019. The p values were calculated by chi-square test.

**FIGURE 1:**
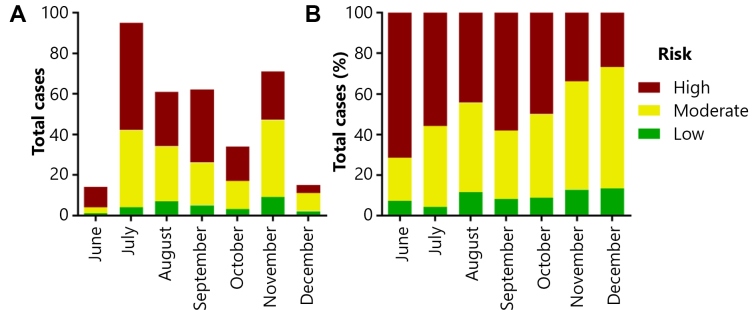
Absolute (A) and relative (B) number of positive cases in each month according to the presumed level of exposure of 1077 HCWs tested for SARS-CoV-2 in Curitiba, Brazil, from June 16, 2020 to December 9, 2020.

Three departments had more than 40% positive infection rates for the evaluated HCWs: semi-intensive care units (17/34, 50%), COVID-19 units (89/187, 47.6%), and surgical units (36/80, 45%). Since the COVID-19 symptom spectrum is broad, from typical respiratory signs to decompensation of underlying diseases, it is worth emphasizing the importance of performing screening tests in all hospitalized patients.

This study had some limitations. First, a negative RT-PCR result should not be used as the only criterion for treatment or patient management decisions, since it does not exclude the possibility of COVID-19 infection^14^. Due to the virus-specific diagnostic window and the evidence that virus shedding may still occur at undetectable levels in the early and late phases of the SARS-CoV-2 infection, the RT-PCR test results should always be interpreted within a broader context^15^. In addition, preanalytical (inadequate collection, handling, transport, or storage of the specimen) and analytical (as active viral recombination or instrument malfunctioning) factors may also compromise the accuracy of RT-PCR detection^14^ ^,^ ^15^. Therefore, a proportion of HCWs with asymptomatic infection may have been missed during the study due to false-negative results. Second, the data obtained relied heavily on participants' self-reports during attendance and were collected retrospectively from clinical records and may have been lost. Finally, we were unable to investigate the disease severity or duration of COVID-19 because hospitalization information was not available in the occupational health clinic records.

However, the present data are crucial to alert administrators and politicians of the need for immediate implementation of measures to reduce the infection rate of these professionals, under the risk of a meaningful reduction in this workforce, which is fundamental during a pandemic.

A total of 32.7% of the HCWs tested positive for COVID-19 in a tertiary hospital in Curitiba, Brazil. The high prevalence of infection among health professionals results in a loss of workforce and strain being placed on healthcare systems. Although HCWs face an increased risk of SARS-CoV-2 transmission while assisting COVID-19 positive patients, transmission may also occur in non-medical areas. The importance of early detection and isolation of infected HCWs, both symptomatic and asymptomatic, is highlighted, which can contribute to the prevention and control of SARS-CoV-2 transmission, both in the hospital and in the community.
